# Implementation of Hemispherical Resonator Gyroscope with 3 × 3 Optical Interferometers for Analysis of Resonator Asymmetry

**DOI:** 10.3390/s22051971

**Published:** 2022-03-02

**Authors:** Myeongseop Kim, Bobae Cho, Hansol Lee, Taeil Yoon, Byeongha Lee

**Affiliations:** School of Electrical Engineering and Computer Science, Gwangju Institute of Science and Technology, 123 Cheomdangwagi-ro, Buk-gu, Gwangju 61005, Korea; mskim2019@gm.gist.ac.kr (M.K.); bobae0413@gm.gist.ac.kr (B.C.); hslee408@gm.gist.ac.kr (H.L.); taeil021@gist.ac.kr (T.Y.)

**Keywords:** hemispherical resonator gyroscope, 3 × 3 optical interferometer, precision vibration measurement, asymmetric hemispherical resonator

## Abstract

A hemispherical resonator gyroscope (HRG) has been implemented by using a consumer wineglass as the resonator and 3 × 3 optical interferometers as the detectors. The poorness of the off-the-shelf wineglass as the resonator can be overcome by the high performance of the optical interferometer. The effects of asymmetries in stiffness and absorption of the resonator are analyzed theoretically and confirmed experimentally. We prove that the trace of the amplitude ratio of two *n* = 2 fundamental resonant modes of the resonator follows a straight line in a complex plane. By utilizing the straightness of the ratio and the high performance of the optical interferometer, we extract four real constant parameters characterizing the HRG system. Experimentally, by using a resonator having an average resonance frequency of 444 Hz and Q value of 1477.2, it was possible to measure the Coriolis force at the level of industrial grade. The bias stability was measured as small as 2.093°/h.

## 1. Introduction

A Hemispherical Resonator Gyroscope (HRG) detects the angular rotation rate by measuring the resonant vibration of a hemispherical resonator. It has the advantages of small size, low noise, high performance, and no wear-out [1]. When a rotation is applied to the HRG, the Coriolis force induced by the rotation changes the vibration pattern of the resonator. Inversely, the Coriolis force can be calculated by measuring the change in the vibration pattern of the resonator. In order to improve the performance of HRG, many studies have been actively performed, mainly with the resonator itself [2,3,4,5,6], the driving method [7,8,9,10,11,12], and the signal processing [13,14,15,16,17]. However, the research on the vibration measurement method has been relatively insufficient.

In order to measure the vibration of the resonator of an HRG system, a device capable of measuring small displacements in a non-contact manner is required. Any contact on the resonator disturbs the vibration of the resonator. In general, capacitive sensors [1,4,18], Laser Doppler Vibrometers (LDV) [18], and optical interferometers [18,19,20] have been used. Capacitive sensors have the advantages of being small, simple, and inexpensive so they are usually used for industrial products [1]. In order to get a high-resolution sensing, the capacitive sensor, with a large area and a narrow gap between electrodes, is needed in general, but for getting a wide dynamic range the sensor having a small area and a wide gap is necessary. There is a tradeoff between the resolution and the dynamic range of the sensor. Although LDV has the highest accuracy, it has a high cost and a large size, so it is widely used as the instrument in the laboratory. Optical interferometers have the advantages of high accuracy and low electromagnetic interference compared to the capacitive sensors. In principle, they are basically simple, cheap, and small compared to LDVs. Several efforts have been made to apply the interferometers to HRG. In particular, the self-mixing interferometer (SMI) has been in the mainstream [18,19,20]. The light emitted from a laser cavity and reflected from the object under measurement re-enters the laser cavity and makes interference with the light within the laser cavity. The vibration of the object can be monitored by measuring the intensity variation, caused by the self-mixing of the laser output. With the SMI, vibration measurement of a sub-micrometer resolution is possible. However, it has the disadvantage of being vulnerable to temperature change [19].

An optical fiber type 2 × 2 directional coupler has been widely utilized as an efficient and simple optical interferometer. It is similar to a Michelson interferometer but has an extended beam splitter, which is simply made by partially splicing two single mode fibers side by side. However, it has a critical problem in practical usage. For small displacement measurements, its sensitivity becomes dependent on the operating point, and is thus highly sensitive to environmental changes, including temperature. Recently, a 3 × 3 optical interferometer has been utilized as a non-contact and high precision measurement device [21]. By adding one more waveguide channel to the conventional 2 × 2 interferometer, the 3 × 3 interferometer gets the signal in the IQ (in-phase and quadrature) mode, which allows for a precision measurement independent of the operating point.

In this paper, the HRG system implemented with a real wineglass and 3 × 3 optical interferometers are presented. For the proof of idea, a consumer wineglass is utilized as a resonator. The resonator is actuated at a point without feedback control to activate the *n* = 2 resonant vibration modes, which has the advantages of being simple to use and cost effective [22]. The vibration pattern of the resonator is optically measured by using two interferometers. By utilizing the ability of complex signal measurement of the 3 × 3 optical interferometer, the complex amplitudes of the two vibration modes of the resonator are measured at the same time. By taking the ratio of the two complex amplitudes, the rotating rate applied on the HRG is calculated. It is well known that the ratio of the normal mode amplitudes is changed with the rotating rate due to the Coriolis force. However, we prove that the trajectory of the mode ratio forms a linear curve in a complex plane even with asymmetries in the resonator structure and in the vibration absorption. By utilizing the high precision measurement ability of the 3 × 3 optical interferometer, the effects of asymmetries in the resonator stiffness and the absorption are extracted first. Secondly, an algorithm that can minimize or compensate for the unwanted effects of the resonator asymmetry on HRG performance is proposed. Finally, we present the proof of the idea that the rotating rate applied to the HRG can be calculated simply by locating the amplitude ratio along the linear curve characterizing the resonator in the complex plane.

## 2. Methods

### 2.1. 3 × 3 Optical Interferometer

**Figure 1:**
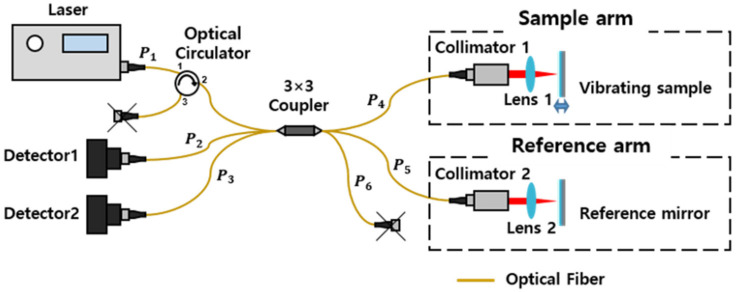
Schematic of a 3 × 3 optical interferometer system. The lights reflected at the sample and the reference arms make interference at the coupler, which is then measured by two detectors simultaneously.

The 3 × 3 optical interferometer having 3 input ports and 3 output ports is a kind of Michelson interferometer. It is simple and low-cost but can be utilized as a high-precision and non-contact sensor device. Park et al. conducted an experiment to measure vibrations of a maximum 1.5 nm displacement amplitude with a 3 × 3 optical interferometer and achieved standard deviation (STDEV) of 0.4 nm without any filtering process [21]. Figure 1 is the schematic of a 3 × 3 optical interferometer configured for measuring a vibrational motion at a point. It can measure both the amplitude and the phase of vibration by using the inherent phase difference among the output return ports of the 3 × 3 coupler.

A laser beam incident to $P_{1}$ is divided into $P_{4}$, $P_{5}$, and $P_{6}$ by the coupler. The lights that went to $P_{4}$ and $P_{5}$ are reflected from the sample arm and the reference arm, respectively, and then make interference to each other at the coupler. The interference signal goes to $P_{1}$, $P_{2}$, $P_{3}$ with the inherent phase difference of the 3 × 3 coupler. The light that goes to $P_{6}$ is not used. The signal returned to $P_{1}$ is also not used, but dumped out using a circulator. The two interference signals going to $P_{2}$ and $P_{3}$ are measured by detectors 1 and 2 at the same time.

In general, the interference signal $I_{x}$, measured at $P_{2}$ port, can be expressed as

(1) $$I_{x}=h+a\cos\phi$$

where, constant h corresponds to DC-offset and a to AC-amplitude. The phase ϕ is the result of interference between the lights of reference and sample arms, and given by

(2) $$\phi =2k_{0}\Delta z+\phi_{0}$$

with the wave-vector magnitude $k_{0}$ and the optical path-length difference Δz between two arms. The additional term $\phi_{0}$ is the initial phase of the interferometer including the inherent phase shift caused by the coupler. For this interference signal $I_{x}$, the second interference signal $I_{y}$, measured at $P_{3}$ port, can be expressed simply as

(3) $$I_{y}=g+b\cos\left(\phi +\delta \right)$$

where, constant g is the DC-offset and b the AC-amplitude, and δ corresponds to the inherent phase difference between two output channels.

With some mathematical manipulations, we can have the phase of the interference signal with a function of the two port measurements, $I_{x}$, $I_{y}$, and the inherent phase difference δ of the 3 × 3 optical interferometer as [21]

(4) $$\tan\phi =\frac{b\left(I_{x}-h\right)\cos\delta -a\left(I_{y}-g\right)}{b\left(I_{x}-h\right)\sin\delta }$$

### 2.2. Analysis of Symmetry Problems in a Hemispherical Resonator

For a simple analysis, the hemispherical resonator is modeled with a pair of oscillators, having lumped elements and coupled with the Coriolis force at Section 2.2.1. The equation of motion of the symmetric resonator is analyzed at Section 2.2.2, and used to show that the amplitude ratio of two fundamental modes is linear with the applied angular rate. The same analysis is performed for an asymmetric resonator at Section 2.2.3, which shows that the trace of the amplitude ratio is not linear with the angular rate any longer but still forms a straight line in a complex plane. By utilizing the linear character of the ratio trace, how to extract the angular rate with an asymmetric resonator is presented in Section 2.2.4.

#### 2.2.1. Lumped Model of a Hemispherical Resonator

In order to measure the Coriolis force, thus the applied rotation, by using the hemispherical resonator, the vibration of the resonator must be measured. However, the asymmetry in the resonator changes the vibrating motion of the resonator. Therefore, it is necessary to analyze and compensate the asymmetry of the resonator as fine as possible. The asymmetry originates mainly from the non-uniformity in the distribution of the shape, density, and composition of the resonator [23]. There are two types of asymmetricity in the hemispherical resonator, stiffness and damping in general. The stiffness asymmetry means that the elasticity of a resonator depends on the position along the lip of the resonator. Similarly, the damping asymmetry means that the degree of damping depends on the vibrating direction.

**Figure 2:**
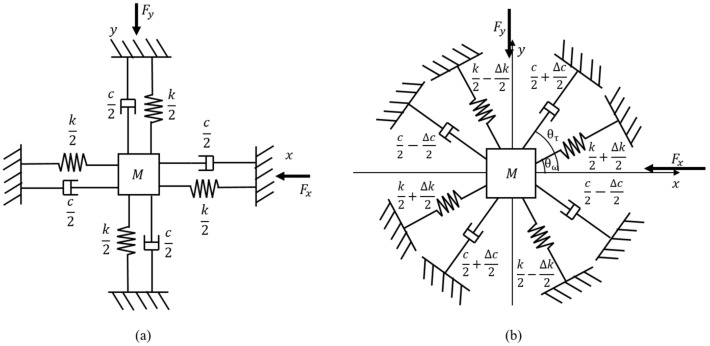
Lumped element models of hemispherical resonators; (**a**) ideal symmetric resonator model, and (**b**) general resonator model having asymmetry in stiffness and damping. *M*: mass, *c*: damping constant, Δ*c*: damping constant difference, $\theta_{\tau }$: damping axis angle, *k*: spring constant, Δ*k*: spring constant difference, $\theta_{\omega }$: spring axis angle, $F_{x}$: driving force in *x* direction, $F_{y}$: driving force in *y* direction.

A hemispherical resonator can be understood as two simple harmonic oscillators coupled by the Coriolis force induced by the rotation. Each oscillator is modeled as having lumped spring, damper, and oscillating mass [24] (p. 55). Figure 2a shows a model of an ideal symmetric resonator. It is noted that the *x*-axis and *y*-axis in the lumped model correspond to the two principal axes, having a relative angle of 45°, of a real hemispherical resonator. The main characteristic of the symmetric resonator is that the spring constant *k* and the damping constant *c* are not dependent on the orientation of axis. Thus, the principal axes are always collinear to the directions of the applied forces, $F_{x}$ and $F_{y}$ as shown in Figure 2a.

Figure 2b shows a model of a general hemispherical resonator having both asymmetries in elasticity and damping. It is modeled as if the spring constants of the two spring axes and the damping constants of the two damper axes are different, and the principal axes are tilted by $\theta_{\omega }$ and $\theta_{\tau }$ against the *x*-axis where the driving force $F_{x}$ is applied.

#### 2.2.2. Ideal Symmetric Resonator

For the symmetric ideal case as shown in Figure 2a, when the driving forces are applied to *x* and *y* axes under a constant angular rotation of a rate $\Omega_{z}$, the motion of the mass can be expressed with two coupled differential equations as [24] (p. 11)

(5) $$M\frac{d^{2}x}{dt^{2}}-2M\Omega_{z}\frac{dy}{dt}+c\frac{dx}{dt}+kx=F_{x}\;$$

(6) $$M\frac{d^{2}y}{dt^{2}}+2M\Omega_{z}\frac{dx}{dt}+c\frac{dy}{dt}+ky=F_{y}\;$$

To get the equations, we assumed that the angular rotation of the system was very slow compared to the vibration frequency of the resonator $\left(\Omega_{z}\ll \omega \right),$ and the rotation rate was constant without acceleration $\left({\dot{\Omega }}_{z}=0\right)$, so that the contribution of the higher order inertial terms and the angular acceleration-related terms could be dropped.

It is assumed that a driving force is applied only along the *x* axis and the force is varying harmonically with a constant frequency ω and a constant amplitude $F_{0}$. In a steady state, the resonant-induced displacements of the resonator in both coordinate axes are simply given with the harmonic signals having the same vibrating frequency as the driving force,

(7) $$x=x_{0}e^{j\left(\omega t+\theta_{x}\right)},\;\;\;\;y=y_{0}e^{j\left(\omega t+\theta_{y}\right)}$$

with $F_{x}=F_{0}e^{j\omega t}$ and $F_{y}=0$. Where the amplitudes $F_{0}$, $x_{0}$ and $y_{0}$ are real values. Substituting Equation (7) into Equations (5) and (6) and doing some arithmetic manipulations give the complex amplitude along each axis as

(8) $$x_{0}e^{j\theta_{x}}=\frac{-M\omega^{2}+jc\omega +k}{{\left(-M\omega^{2}+jc\omega +k\right)}^{2}-4M^{2}\Omega_{z}{}^{2}\omega^{2}}F_{0}$$

(9) $$y_{0}e^{j\theta_{y}}=-\frac{j2\Omega_{z}\omega }{{\left(-M\omega^{2}+jc\omega +k\right)}^{2}-4M^{2}\Omega_{z}{}^{2}\omega^{2}}F_{0}$$

By taking the ratio of Equation (9) to Equation (8), we have the relationship of

(10) $$\frac{y}{x}=\frac{y_{0}}{x_{0}}e^{j\left(\theta_{y}-\theta_{x}\right)}\equiv R_{0}e^{j\theta }=\frac{-j2M\omega }{-M\omega^{2}+jc\omega +k}\Omega_{z}\equiv A\Omega_{z}$$

It says that the vibration ratio is proportional to the angular rate $\Omega_{z}$, regardless of the amplitude of the driving force. The proportional constant *A* is a complex number and determined by the resonator parameters only. Thus, with the resonator parameters, obtained before the main measurements, the applied angular rate can be extracted by measuring the complex amplitudes along both coordinate axes.

As was discussed with Equation (10), the amplitude ratio *y*/*x* corresponds to a point along the line directed by the complex constant *A* in a complex plane. For the ideal symmetric resonator case, the line is passing through the origin of the coordinates; the ratio has a zero length, $R_{0}=0$, at the zero angular rate, $\Omega_{z}=0$. Experimentally, the complex constant *A*, characterizing the resonator, can be obtained by measuring the ratio of Equation (10) for at least two known $\Omega_{z}$’s.

#### 2.2.3. General Asymmetric Resonator

Unlike the ideal symmetric resonator, a general hemispherical resonator has asymmetries in stiffness and damping. The non-uniform stiffness, or elasticity, of the resonator around the lip can be counted by introducing the offset angle $\theta_{\omega }$ between the driving force direction *x* and the principal axis of the elasticity as shown in Figure 2b. Of course, the net spring constants representing the two orthogonal normal modes of the resonator are set differently by Δk to each other. The same thing can be assumed with the asymmetric damping of the resonator. For this more general resonator, the equation of motion of the lumped model is given as [24] (p. 17)

(11) $$\begin{array}{c} M\frac{d^{2}x}{dt^{2}}-2M\Omega_{z}\frac{dy}{dt}+c\frac{dx}{dt}+\Delta c\left\{\frac{dx}{dt}\cos\left(2\theta_{\tau }\right)+\frac{dy}{dt}\sin\left(2\theta_{\tau }\right)\right\}+kx\\ +\Delta k\left\{x\;\cos\left(2\theta_{\omega }\right)+y\sin\left(2\theta_{\omega }\right)\right\}=F_{x} \end{array}$$

(12) $$\begin{array}{c} M\frac{d^{2}y}{dt^{2}}+2M\Omega_{z}\frac{dx}{dt}+c\frac{dy}{dt}+\Delta c\left\{\frac{dx}{dt}\sin\left(2\theta_{\tau }\right)-\frac{dy}{dt}\cos\left(2\theta_{\tau }\right)\right\}+ky\\ +\Delta k\left\{x\sin\left(2\theta_{\omega }\right)-y\cos\left(2\theta_{\omega }\right)\right\}=F_{y} \end{array}$$

With the single applied force, same as the ideal symmetric case of $F_{x}=F_{0}e^{j\omega t}$ and $F_{y}=0$, the vibrations in a steady state are calculated as

(13) $$\begin{array}{c} x_{0}e^{j\theta_{x}}=\\ \frac{-M\omega^{2}+j\left\{c-\Delta c\;\cos\left(2\theta_{\tau }\right)\right\}\omega +k-\Delta k\;\cos\left(2\theta_{\omega }\right)}{{\left(-M\omega^{2}+jc\omega +k\right)}^{2}-{\left(j\Delta c\omega +\Delta k\right)}^{2}+2j\Delta c\omega \Delta k\left\{1-\cos\;\left(2\theta_{\tau }-2\theta_{\omega }\right)\right\}-4M^{2}\Omega_{z}{}^{2}\omega^{2}}F_{0} \end{array}$$

(14) $$\begin{array}{c} y_{0}e^{j\theta_{y}}=\\ \frac{-j\left\{2M\Omega_{z}+\Delta c\sin\left(2\theta_{\tau }\right)\right\}\omega -\Delta k\;\sin\left(2\theta_{\omega }\right)}{{\left(-M\omega^{2}+jc\omega +k\right)}^{2}-{\left(j\Delta c\omega +\Delta k\right)}^{2}+2j\Delta c\omega \Delta k\left\{1-\cos\;\left(2\theta_{\tau }-2\theta_{\omega }\right)\right\}-4M^{2}\Omega_{z}{}^{2}\omega^{2}}F_{0} \end{array}$$

Taking the ratio between Equations (13) and (14) gives

(15) $$\frac{y_{0}}{x_{0}}e^{j\left(\theta_{y}-\theta_{x}\right)}=-\frac{j\left\{2M\Omega_{z}+\Delta c\sin\left(2\theta_{\tau }\right)\right\}\omega +\Delta k\;\sin\;\left(2\theta_{\omega }\right)}{-M\omega^{2}+j\left\{c-\Delta c\cos\left(2\theta_{\tau }\right)\right\}\omega +k-\Delta k\;\cos\;\left(2\theta_{\omega }\right)}\;$$

Similar to the symmetric case, we can set this complex ratio as

(16) $$\frac{y}{x}=\frac{y_{0}}{x_{0}}e^{j\left(\theta_{y}-\theta_{x}\right)}\equiv Be^{j\theta }\left\{j\left(\Omega_{z}+a\right)+b\right\}$$

where the real constants *B*, θ, *a*, *b* are defined as;

(17) $$Be^{j\theta }=-\frac{2M\omega }{-M\omega^{2}+j\left\{c-\Delta c\cos\left(2\theta_{\tau }\right)\right\}\omega +k-\Delta k\;\cos\;\left(2\theta_{\omega }\right)}$$

(18) $$a=\frac{\Delta c\sin\left(2\theta_{\tau }\right)}{2M}$$

(19) $$b=\frac{\Delta k\;\sin\;\left(2\theta_{\omega }\right)}{2M\omega }$$

**Figure 3:**
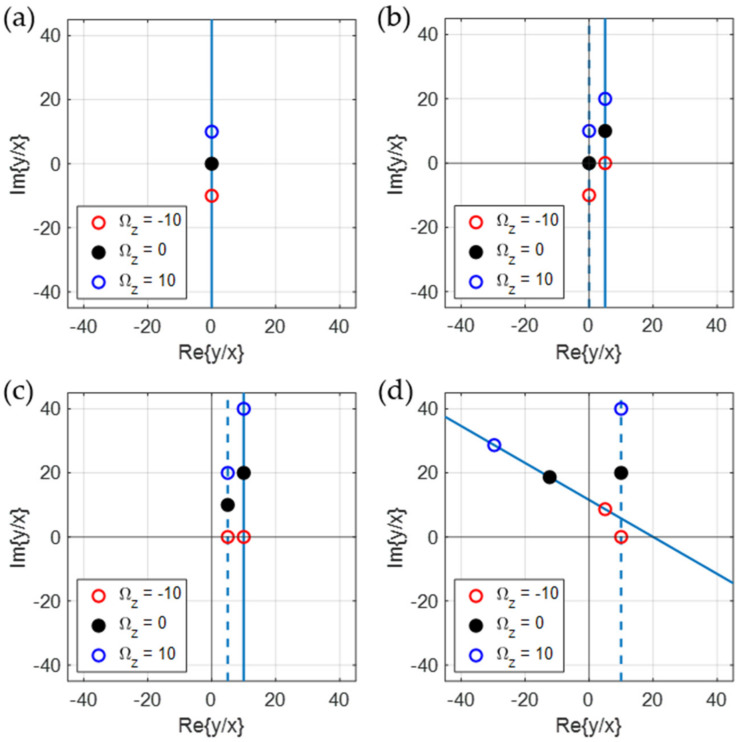
The trace of the ratio *y/x* in Equation (16) simulated with various resonator parameters and drawn in a complex plane. The trace is plotted when *B*, θ, *a*, and *b* are (**a**) 1, 0°, 0, and 0; (**b**) 1, 0°, 10, and 5; (**c**) 2, 0°, 10, and 5; (**d**) 2, 60°, 10, and 5, respectively. The trace is always on a straight line and the line is determined by the four real constants *B*, θ, *a*, and *b* of Equation (16). The dotted line is the trace made with just the previous conditions.

Interestingly, we can prove that the trace of the ratio *y/x* in Equation (16), plotted in terms of $\Omega_{z}$, forms a straight line in a complex plane. In the equation, the factor $\left\{j\left(\Omega_{z}+a\right)+b\right\}$ means that the vertical line of $\Omega_{z}$ in Figure 3a is shifted to the horizontal direction by *b* and the point of $\Omega_{z}=0$ moves along the vertical direction by *a* as shown with Figure 3b. Furthermore, the shifted line is magnified by *B*, which shifts the line to the horizontal direction and extends the mutual distance between two points on the line as shown with Figure 3c. Finally, as in Figure 3d, the line is rotated by an angle θ with the factor of $e^{j\theta }$. Since Equation (18) is related to the damping asymmetry and Equation (19) with the stiffness asymmetry, it can be understood that the constants *a* and *b* represent the effects of damping asymmetry and stiffness asymmetry on the vibration of the resonator, respectively. Figure 3a is drawn with (*B*, θ, *a*, *b*) = (1, 0, 0, 0), Figure 3b with (1, 0, 10, 5), and Figure 3c with (2, 0, 10, 5). Finally, Figure 3d drawn with (*B*, θ, *a*, *b*) = (2, 60°, 10, 5) shows a straight line inclined and not passing through the origin of the coordinates. In the final figure, we can see that the $\Omega_{z}=0$ point is not at the origin of the coordinates (due to *b* ≠ 0) and the distance along the line for a given ${\Delta \Omega }_{z}$ (for an example, between the points of $\Omega_{z}=0$ and $\Omega_{z}=-10$) is enlarged (due to *B* > 1).

#### 2.2.4. Extracting the Applied Angular Rate with Asymmetric Resonator

In the symmetric resonator, the magnitude of the ratio in Equation (10) was linearly proportional to the angular rate applied to the system. However, for the asymmetric resonator case, the ratio in Equation (16) is not linearly proportional to $\Omega_{z}$ any longer due to the stiffness asymmetry and the damping asymmetry as shown in Figure 3. However, the linear character of the ratio trace in a complex plane allows us to extract the applied angular rate even with the asymmetries in resonator. Mathematically, the angular rate can be derived from Equation (16) as

(20a) $$\Omega_{z}=j\left(b-\frac{y/x}{Be^{j\theta }}\right)-a$$

or, by using the real character of $\Omega_{z}$, *a*, and *b*, it can be expressed as

(20b) Ωz=Imy/xBejθ−a

The equation says that, at least in principle, the applied angular rate $\Omega_{z}$ can be extracted from the complex ratio y/x. The four real constants (*B*, θ, *a*, *b*), characterizing the resonator of an HRG system can be obtained by calibrating the system with several known $\Omega_{z}$’s. For the best case, two complex measurements are enough to get the four real constants to compose a straight line in a complex plane.

**Figure 4:**
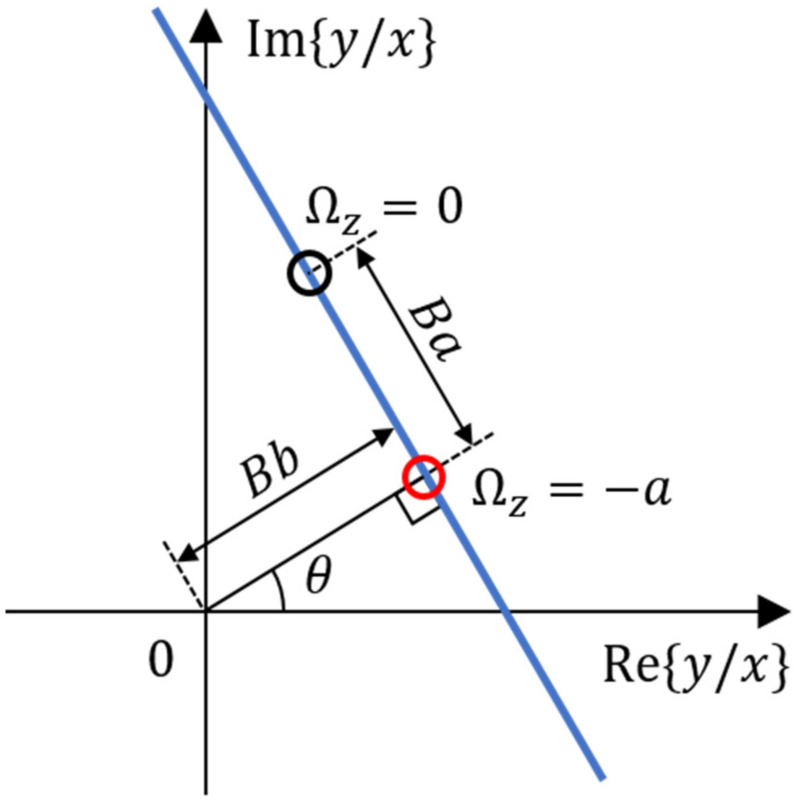
Analysis of the *y/x* trace for an asymmetric resonator. The *y/x* ratios collected with various angular rates form a straight line in a complex plane. The plot shows that the distance from the origin to the nearest point on the line is *Bb*, and the angular rate giving the nearest point is −*a*, and the distance from the nearest point to the $\Omega_{z}=0$ point, along the line, is *Ba*.

The principle for getting Equation (20) can be a little more deeply understood with Figure 4, the same one as Figure 3d. For plotting the straight line in the figure, the first thing is taking the best fitting linear curve with the complex ratios of Equation (16) measured at various angular rates. Then, the distance from the origin of the coordinates to the nearest point on the line is calculated as the value of *Bb*. The angular rate giving the nearest point to the origin is interpolated as the value of −*a*. Furthermore, the distance from the nearest point to the point obtained with $\Omega_{z}=0$ is calculated as the value of *Ba*. The angle of the line against the vertical line is the angle θ in Equation (16). By utilizing this process, all constants of Equation (16) or Equation (20) can be obtained. Even though it would be tough in calculation, the vibration measurements made at any other two different but known angular rates can be enough to determine the 4 constants for calibration. In order to perform the calibration effectively, it is necessary to precisely measure the complex amplitude of vibration. Not only the magnitude but also the phase of the interference signal are necessary. Therefore, the high resolution of the 3 × 3 interferometer and its IQ measurement ability increase the accuracy in the angular rate extraction with the HRG having a rather poor resonator.

## 3. Experiments

### 3.1. Hemispherical Resonator

**Figure 5:**
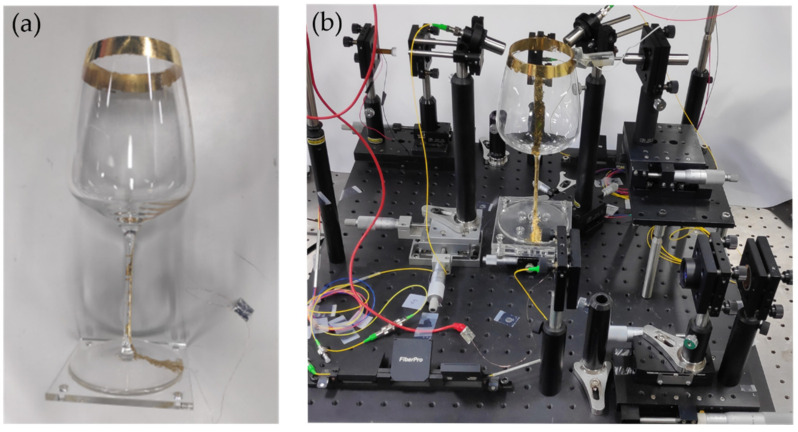
The photographs of (**a**) the hemispherical resonator and (**b**) the rotating part of the implemented HRG system. A general consumer wineglass was used as the resonator. A sheet of thin gold foil was attached and used as the electrode for activating the resonator.

As the resonator of HRG, a general consumer wineglass was used as shown in Figure 5. The frequencies of two resonant modes of the wineglass, off the shelf, were different by as much as 4 Hz to each other. To match the resonant frequencies, and thus to increase the coupling efficiency between the two resonant modes, a part of the wineglass lip was mechanically grinded little by little. It allowed us to have the resonance frequency difference as small as 0.3 Hz; 444.1 Hz for the primary mode and 443.8 Hz for the secondary mode. In addition, in order to activate electrostatic force for vibrating the resonator, as an electrode, a sheet of thin gold foil (Allgoldleaf, Sangjabio Co., Ltd., Daegu, Korea) was attached to the resonator surface with gelatin. The resonator was activated in air without using a vacuum chamber.

Even though it is well known, it is noted that the flexural vibration of the lip of a wineglass is dominantly in a shape of an ellipse. The displacement of the lip from its averaged circle line is sinusoidal and has two antinodes along a round trip, called the *n* = 2 fundamental flexural resonant mode. There are two *n* = 2 fundamental modes, called primary and secondary, and in general their principal axes are separated by 45° to each other. By taking a linear combination of these two fundamental modes, any *n* = 2 vibrating motion of the lip can be described. When the wineglass is under rotation, the node line of the vibrating motion follows the rotation with a lag due to the Coriolis force. For the case of ideal symmetric resonator, the fundamental modes are degenerated, so that the principal axis of the primary mode can have any direction and the resonant frequencies are equal to each other. However, for the elastically asymmetric case, the resonant frequency splits and depends on the direction of the node line. The mode having the higher frequency is called the primary mode and the lower one the secondary mode.

At a point on the wineglass, the resonant frequencies were searched by scanning the deriving frequency, which gave maximum vibration amplitudes. While rotating the glass and deriving the resonator at the maximum resonant frequency, the principal axis of the primary mode was found as the one giving the minimum vibration amplitude of the secondary mode. In experiments, the resonator was activated along the principal axis of the primary mode with its resonant frequency, 444.1 Hz. The Q value of the resonator was obtained by measuring the decay time of each resonant mode. For the primary and secondary modes, the Q values were 1529.67 and 1424.76, respectively.

### 3.2. Experimental Setup

**Figure 6:**
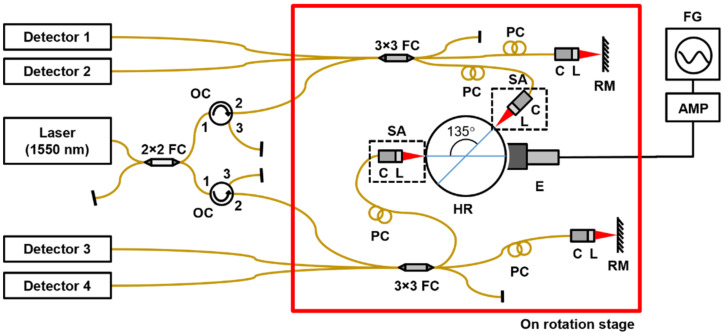
The HRG system implemented with 3 × 3 optical interferometers. The system operates in the non-feedback open-loop (NFOL) mode. RM: reference mirror, SA: sample arm, PC: polarization controller, HR: hemispherical resonator, E: electrode, FC: fiber coupler, OC: optical circulator, FG: function generator, AMP: amplifier, C: collimator, L: lens.

Figure 6 shows the schematic of the proposed system. For activating the resonator, the non-feedback open-loop (NFOL) mode is used, in which the driving force is applied only to the principal axis of the primary mode without feedback control. A uniform sinewave of a 0~220 V magnitude is applied to the electrode to generate the electrostatic force. The angular rate, to be measured by the system, is applied by rotating the optical table having the components within the red box of Figure 6 with a motor.

To measure the vibrations of two fundamental resonant modes of the resonator, two 3 × 3 optical interferometers are used. Light comes out from a 1550 nm laser and splits in two. One light enters the interferometer that measures the primary mode oscillating along the driving axis, and the other light enters the other interferometer for the secondary mode. Even though the node lines of two fundamental resonant modes are apart from each other by 45°, due to space limitations the first sensing probe is placed at the opposite side of the force driving point.

## 4. Results

### 4.1. Measurements of Vibrations of Two Fundamental Resonant Modes

**Figure 7:**
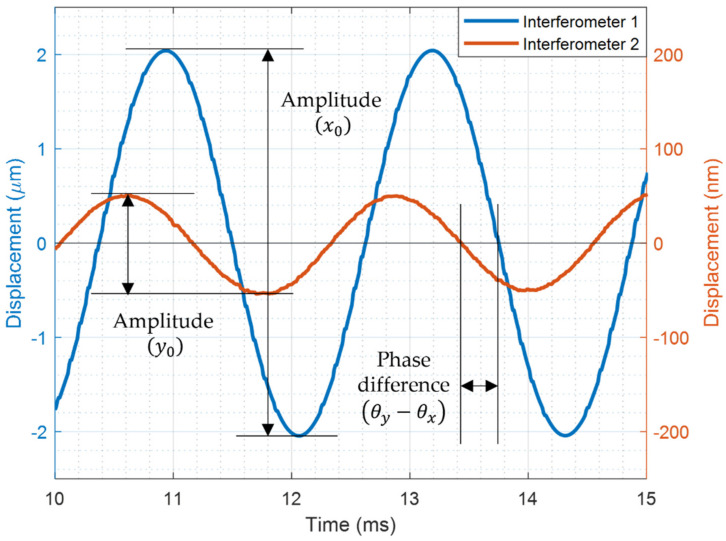
The displacements induced by the vibrations of two fundamental modes of a hemispherical resonator derived by a single actuator. The system was rotated by a constant rate of $\Omega_{z}=-4.19{}^{\circ}/s$. A phase difference of 61.8° between two modes was measured.

Figure 7 shows the displacements induced by the vibration of a hemispherical resonator, measured at the same time with two 3 × 3 optical interferometers along two coordinate axes. In general, for the case of small coupling, the vibration amplitude $\left(x_{0}\right)$ in the *x* direction along which the driving force is applied is larger than the amplitude $\left(y_{0}\right)$ in the *y* direction. In the figure, we can see that one mode is very large and the other is very small in amplitude. It shows that small amount of the primary mode energy was coupled to the secondary mode due to the Coriolis force. The rotation rate applied for this result was $\Omega_{z}=-4.19{}^{\circ}/s$. Furthermore, we can see that the two modes are not matched in phase; there is a phase difference of 61.8° between them. As was discussed, the amplitudes ($x_{0}$, $y_{0}$) and the phase difference $\left(\theta_{y}-\theta_{x}\right)$ of two fundamental modes must be measured to extract the applied angular rate. It is noted that as a displacement sensor, the 3 × 3 optical interferometer measures the vibration of a hemispherical resonator in real time.

### 4.2. Angular Rotation Rate Extraction

**Figure 8:**
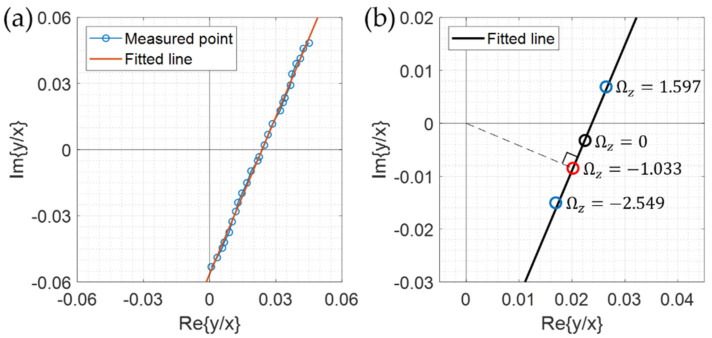
The experimental results: (**a**) the plot of amplitude ratios *y/x* of Equation (20) measured with various angular rates, and (**b**) the fitted straight line characterizing the resonator. From the fitting with a straight line, the 4 real constants characterizing the resonator are extracted as *B* = 0.318, θ = −22.84°, *a* = 1.033°/s, and *b* = 3.944°/s.

**Figure 9:**
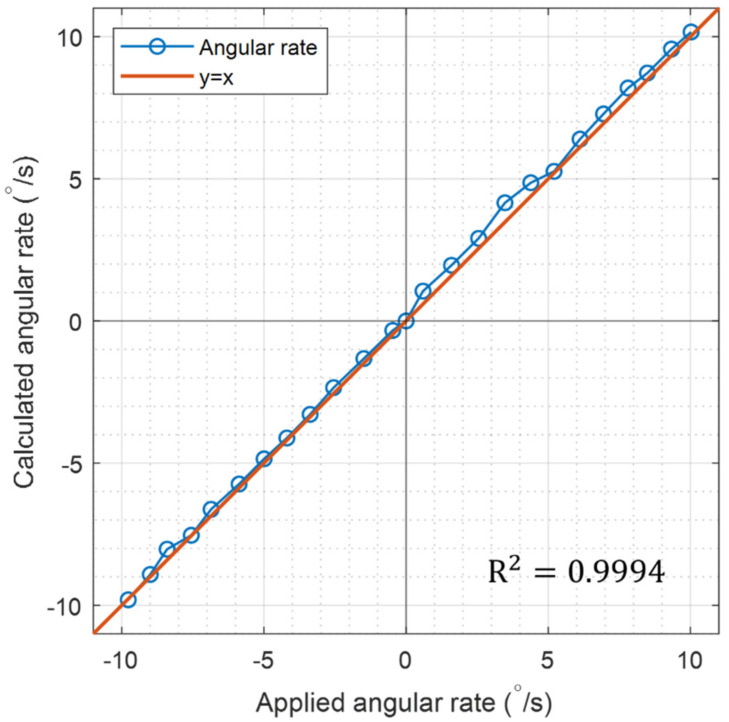
The comparison of the applied angular rate and the measured angular rate. They are well matched with the coefficient of determination of $R^{2}$ = 0.9994.

The angular rotation rate was externally applied to the system for characterizing the resonator by changing the rate step-by-step in a range of −9°/s to 9°/s, and the resulting vibrations of both fundamental modes were measured by two 3 × 3 optical interferometers at the same time. After calculating the ratio of amplitudes of two modes for each applied angular rate, the ratio was located in a complex plane as in Figure 8a. For 25 different rates, the complex ratios were plotted and fitted with a curve. We can see that the data points are well aligned along a straight line. With the process mentioned with Figure 4, the four real constants composing Equation (16) were calculated as *B* = 0.318, θ = −22.84°, *a* = 1.033°/s, *b* = 3.944°/s, and the resulted straight line was depicted in Figure 8b. With these constants, the angular rate was extracted by using Equation (20) for each applied rate. The rate extraction was tried with the method explained with Figure 4 also, and the same result was obtained. The extracted angular rates were plotted in terms of the known applied rates in Figure 9 and compared. We can see that the data points are well aligned with the *y = x* line with the coefficient of R^2^ = 0.9994, which means that the measured angular rate and the reference one (applied angular rate) are well matched to each other. In other words, we can say that the asymmetries of the rather poor hemispherical resonator have been well counted or compensated with the measurements made with the proposed optical IQ interferometers.

### 4.3. Bias Stability Measurement

As a part of evaluating the performance of the implemented HRG system, bias stability was measured. It is known that the bias stability is related to the minimum detectable angular rate of a gyroscope, which is generally measured by taking the minimum value of the Allan deviation [24] (pp. 7–8). At zero applied angular rate, the rate $\Omega_{z}$ of Equation (20) has been measured for 2 h at every cycle of oscillation of the resonator. The measured angular rate was integrated for an integration time or interval, and then divided by the interval, and the difference between adjacent intervals was taken. By squaring the difference and taking the ensemble average with respect to time, the Allan variance was obtained. Finally, the Allan deviation was calculated by taking the square root of the Allan variance.

**Figure 10:**
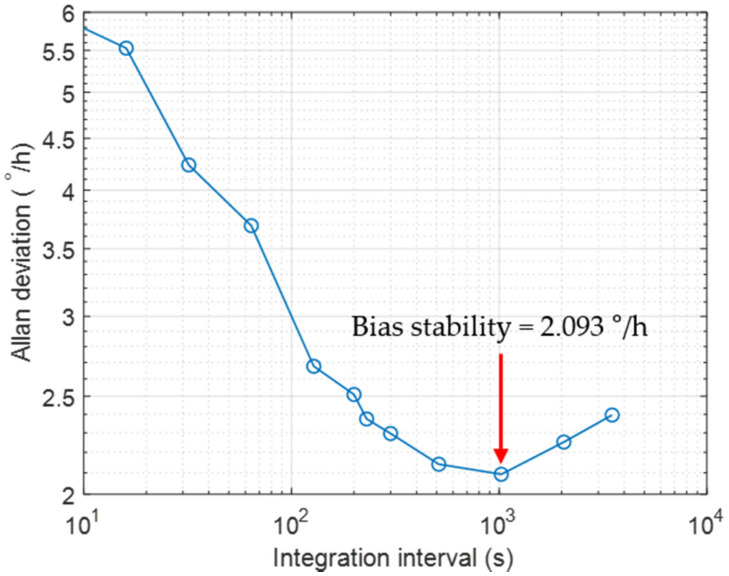
The plot of Allan deviations made with the implemented HRG. The measurements have been made for 2 h at room temperature without temperature control. The bias stability was 2.093°/h, which corresponds to an industrial-grade gyroscope.

Figure 10 is the Allan deviations of the implemented HRG system, which were measured as increasing the integration time interval rather constantly in a log scale. As the bias stability of the system, the minimum value of the Allan deviation plot was taken. The measurements were made at room temperature without temperature control. As a result of the measurements, the bias stability was obtained as small as 2.093°/h, which corresponded to an industrial-grade gyroscope (which is in the range of 1 to 30°/h [25]).

## 5. Discussion

The bias stability of the implemented HRG was measured as 2.093°/h, which corresponded to the industrial grade. This high stability seems to be achieved because of the high precision measurement of the 3 × 3 optical interferometer. It could give not only the amplitude but also the phase of the vibration without being affected by the initial condition of the interferometer.

Because a consumer wineglass was used as the resonator, it was not easy to directly compare the performance of the implemented system with other reported high-end dedicated systems. Even though we have tried to balance the resonator by mechanically grinding the wineglass, there was appreciable asymmetricity not only in stiffness but also in damping. Due to the high volume of the wineglass, operating the system in a vacuum circumstance was practically impossible, which increased the damping and thus hurt the Q value of the resonator. However, with the help of the high precision of the optical interferometer, we could overcome the disadvantages of the asymmetricity of the non-dedicated resonator and achieve the industrial grade performance. For upgrading the system to the tactical grade or higher, it is necessary to operate the system in a vacuum and to use a dedicated resonator having a good symmetry and high Q value. A cavity having high Q value has a long response time in general. Thus, we need to think of feedback control in deriving the resonator.

In Equation (10) for a symmetric resonator, the mass *M* appears at the denominator and numerator at the same time. Thus, dividing both of them with *M* simplifies the equation as

(21) $$\frac{y}{x}=\frac{-j2M\omega }{-M\omega^{2}+jc\omega +k}\Omega_{z}=\frac{-j2\omega }{-\omega^{2}+j\omega c/M+k/M}\Omega_{z}$$

Furthermore, by introducing the average resonant frequency $\omega_{0}\equiv \sqrt{k/M}$ and the decay constant τ≡M/c of the resonator, we have the equation in a more familiar form of

(22) $$\frac{y}{x}=\frac{-j2\omega }{\left(\omega_{0}^{2}-\omega^{2}\right)+j\omega /\tau }\Omega_{z}$$

The similar simplification can be made with Equation (15) for the asymmetric case also.

**Figure 11:**
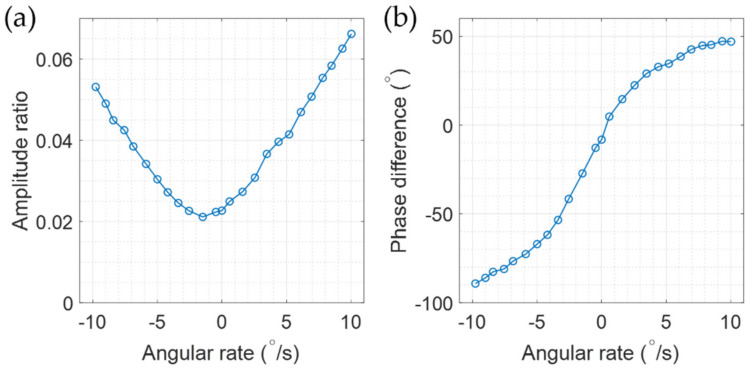
The amplitude ratio *y/x* measured at several angular rates: (**a**) the magnitude and (**b**) the phase of the ratio. The magnitude variation is not linear to the applied angular rate, and does not vanish at any rate. The phase varies with the angular rate but the sign of the phase is not changed exactly at the zero rate.

With Equation (16), we know that the measured ratio *y*/*x* has a complex value. If we take care of only the magnitude of the complex ratio, $\left|y/x\right|$, we encounter some problems in extracting the angular rate. At first, the same ratio happens at two different angular rates, as shown Figure 11a, thus we cannot distinguish the direction of rotation. Secondly, at the zero angular rate the ratio does not have the minimum value. The ratio does not reach zero for any rate. Even worse, the trace is not fitted with a linear curve, but varies nonlinearly. By considering the phase variation of the ratio as shown with Figure 11b, the direction of rotation can be determined by some means. However, the other problems, such as reaching the minimum ratio at a non-zero rate and the nonlinear variation of the ratio with the angular rate, cannot be treated effectively.

Even though the optical measurement is still bulky and expensive, with its high precision and non-contact measurement abilities, it can be used for evaluating or analyzing the resonator of an HRG system. From a technical point of view, we can say that the optical interferometer can be supplied with a low cost and small photonic integrated chip (PIC) in the near future. We can think of installing the optical pickup even in a commercial HRG system. The light can be delivered even to a micro-shell resonator by utilizing micro scale planar waveguides lithographically fabricated on the substrate of the resonator.

The resonant frequency of a high-end hemispherical resonator is in a range of tens of kHz and the Q value is as high as 10^7^ [26,27,28]. The stability of commercial products is as fine as 0.015°/h [1,29,30]. The optical sensing is expected to accelerate, achieving such a high-end performance.

The attachment of the gold film on the wineglass might hurt the SNR of the system. We have tried not to modulate the surface by using nanometer-thick film. However, for a better performance as a resonator, printing the electrodes and connecting wires directly on the glass surface will be better. The variation of the optical reflectance due to the filming might hurt the optical pickup also. The system was configured so that the incident light irradiated the surface perpendicularly. The signal-to-noise was measured as 69.1 dB.

## 6. Conclusions

A HRG (hemispherical resonator gyroscope) system has been implemented with a consumer wineglass and optical interferometers. Even with the many disadvantages of using a non-dedicated resonator, not using a vacuum circumstance and with no feedback control, industrial grade performance could be achieved. It has been analyzed and measured that the trace of the amplitude ratio between two fundamental modes of the resonator formed a straight line in a complex plane even with the resonator’s asymmetries in stiffness and absorption. Along the straight line, it was confirmed that the trace of the complex ratio was linearly proportional to the applied angular rate. With the help of high resolution and the IQ (in-phase and quadrature) measuring ability of a 3 × 3 optical interferometer, the parameters characterizing the resonator could be effectively extracted and successfully used to compensate for the disadvantages of a rather poor resonator.

By mechanically grinding a consumer wineglass, off-the-shelf, the frequency difference between two fundamental resonant modes of the resonator could be made as small as 0.3 Hz; 444.1 Hz and 443.8 Hz. The Q value of each mode was measured as 1529.67 and 1424.76, respectively. The bias stability of the implemented HRG system was measured as small as 2.09°/h, corresponding to the industrial grade. Even though the 3 × 3 optical interferometer is bulky to be directly installed in a practical system, it can be used to analyze resonators and calibrate systems. In the near future, it is expected that a cost-effective and small size interferometer, implemented in the form of photonic integrated chip (PIC), would accelerate the development of a high-end HRG system.

## Data Availability

The data presented in this study are available on request from the corresponding author. The data are not publicly available due to privacy or ethical restrictions.
